# Longitudinal associations between sleep duration and cognitive impairment in Chinese elderly

**DOI:** 10.3389/fnagi.2022.1037650

**Published:** 2022-11-17

**Authors:** Wei-chao Chen, Xiao-yan Wang

**Affiliations:** ^1^School of Journalism and Communication, Hunan Normal University, Changsha, China; ^2^College of Finance and Statistics, Hunan University, Changsha, China

**Keywords:** sleep duration, cognitive impairment, U-shaped association, changes in sleep duration, cognitive changes, Chinese population

## Abstract

**Background:**

Age-associated cognitive decline has become a major threat to both personal welfare and public health and can further develop into Dementia/Alzheimer’s disease. Sleep is significantly correlated with cognitive function, but both cognitive impairment and sleep problems increase with normal aging. This study explored how sleep duration affects cognitive performance among older adults in China.

**Methods:**

Using data from the Chinese Longitudinal Healthy Longevity Survey (CLHLS) in 2014 and 2018, cognitive function was assessed *via* the Mini-Mental State Examination (MMSE), which included five domains: orientation, registration, attention or calculation, recall, and language. Logistic regression was used to examine whether the change in sleep duration was a risk factor for cognitive impairment. We also used multinomial logistic regression to study the impact of sleep duration and the changes in sleep duration on cognitive changes during the follow-up period.

**Results:**

The empirical study showed a U-shaped relationship between sleep duration and increased risk of cognitive impairment. Short (< 6 hours) and long (> 8 hours) sleep durations were positively associated with cognitive impairment. Tests of interactions between sleep duration and sleep quality showed that short sleep durations with fair sleep quality had an increased risk of cognitive impairment. Further, the participants were divided into three groups: normal cognition (MMSE > 24), mild cognitive impairment (MCI, 18 ≤ MMSE score ≤ 24), and severe cognitive impairment (MMSE < 18). First, of the participants with normal cognition at baseline, those who sleeping > 7 h at follow-up and > 7 h at both baseline and 4-year follow-up assessments could increase the risk of cognitive impairment. Second, for individuals with MCI at baseline, those who transitioned to sleeping > 7 h at follow-up period and > 7 h at both baseline and 4-year follow-up assessments had a lower chance of reverting to normal cognition.

**Conclusion:**

Excessive sleep may be a major risk for cognitive impairment among older adults. Furthermore, a moderate amount of sleep could be a possible strategy to prevent cognitive impairment.

## Background

Cognitive ability is the capacity of the human brain to process, store and extract information (Hunter, 1986). Cognitive decline is a progressive neurodegenerative disease associated with increasing age among the elderly (Li et al., 2019; Kioussis et al., 2021; Liu et al., 2021). The aggravation of cognitive decline is likely to lead to Alzheimer’s disease, dementia, and death (Backman et al., 2005; Prince et al., 2015). By 2018, the number of Alzheimer’s patients in China has increased from about 3.68 million in 1990 to nearly 10 million, ranking first in the world (Liu and Guo, 2022), posing huge pressure on informal care costs and health resources. Moreover, the cognitive health of elderly people is a very important part of healthy aging (Mei et al., 2017).

Moderate sleep duration is particularly important for optimal cognitive function. Aging also leads to changes in the sleep patterns of the elderly. And shorter or longer sleep duration was associated with a higher risk of cognitive decline. Multiple studies have assessed longitudinal associations between sleep duration and cognitive function in the elderly. However, to date, the results of these associations have been contradictory. For example, studies have shown that short (6 h or less/day) and long (9 h or more/day) durations of sleep are associated with a risk of cognitive impairment (Kronholm et al., 2009; Virta et al., 2013), whereas other studies did not find a “U-shaped” association. For example, some illustrated that only longer sleep was associated with the risk of poorer cognitive function in the elderly (Faubel et al., 2009; Loerbroks et al., 2009; Ramos et al., 2013). The inconsistent results may be due to differences in methodology and sample data.

Recently, although many studies have started to pay attention to changes in sleep duration, their results are still inconsistent. Several studies found that increased sleep durations at follow-up were associated with greater cognitive decline (Benito-León et al., 2009; Gildner et al., 2019), or all-cause dementia (Benito-León et al., 2009). Hua et al. (2020) found that an increase in sleep duration from short to moderate was significantly associated with better global cognition scores among the Chinese elderly. Ferrie et al. (2011) demonstrated participants who moved from a regular pattern of 6–8 h per night to shorter and longer durations of sleep were associated with poorer cognitive function. Moreover, few studies have focused on the association between changes in sleep duration and cognitive change over time. The absence of baseline cognitive performance data implies that the associations would lead to reverse causality, the participants with baseline cognitive impairment being more likely to develop adverse sleep patterns during the follow-up period (Xu et al., 2014).

Although considerable studies have focused on the associations between sleep duration and cognitive function, the results are still inconsistent. In addition, few studies have tested how changes in sleep duration over time in association with cognitive impairment. Thus, our purpose was to clarify how changes in sleep patterns might impact age-related cognitive deficits among Chinese elderly people. Based on longitudinal data from the Chinese Longitudinal Healthy Longevity Survey (CLHLS) in 2014 and 2018 follow-up data, this current study enriches and expands previous research in three aspects. **Firstly,** several studies targeting Chinese older adults (Gu et al., 2010; Lee et al., 2018; Wang et al., 2020) were based on a cross-sectional design, but we provided a perspective on changes in sleep duration by using a large study sample with longitudinal data. **Secondly,** in comparison with the earlier studies, we evaluated the impact of sleep duration and the changes in sleep duration on cognitive impairment. **Thirdly,** we explored the impact of sleep duration and the changes in sleep duration on cognitive changes over 4 years.

## Materials and methods

### Participants

The data used in the study are selected from a publicly accessible database of the CLHLS, conducted by the Chinese Center for Disease Control and Prevention and Peking University. The baseline survey of the CLHLS program is officially launched in 1998 and followed-up every 2–3 years since, which covers 23 provinces/cities/autonomous regions in mainland China. The CLHLS adopts a multi-stage unequal proportion target random sampling method (Wu et al., 2021), which represents 85% of the total population in China. As a nationwide and comprehensive longitudinal tracking survey, the CLHLS provides high-quality longitudinal data for academic/scientific investigation and policy research in important areas such as Chinese society, population, and health. The present study included individuals who had data in 2014 as a baseline and 2018 as a follow-up for data analysis (the latest datasets we could obtain when this study was carried out).

**FIGURE 1:**
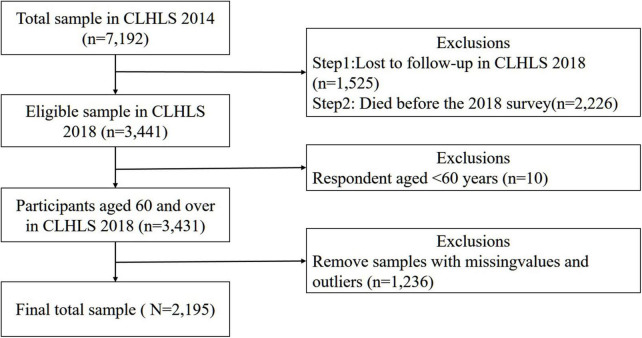
Flowchart of sample selection and preprocessing (*n* represents sample size).

A total number of 15,874 participants were enrolled in CLHLS (2018). We used 2 waves of CLHLS data conducted in 2014 and 2018 to analyze how sleep duration influenced cognitive function. In 2018, 1,525 were lost to follow-up and 2,226 older adults died before the re-interview. After excluding respondents with missing values and under 60 years old, 2,195 out of 7,192 eligible participants were included for analyses. Details about the sample selection and preprocessing are shown in Figure 1.

### Cognitive function

As a classic cognitive function assessment tool, the Mini-Mental State Examination (MMSE) is widely used to assess cognitive function worldwide (Zhang et al., 1990; König et al., 2017). The Chinese versions of the MMSE was developed and validated in Chinese populations (Chen et al., 2021). In CLHLS, the MMSE includes five aspects of cognitive functioning: orientation, registration, attention or calculation, recall, and language. More details about MMSE are shown in Table 1.

**TABLE 1:** Details about the Chinese version of the modified mini-mental state examination.

Dimensions	Item	Scores
Orientation	1. What time of day is it now (morning, afternoon, evening)?	1
	2. What is the animal year of this year?	1
	3. What is the date (day and month) of the mid-autumn festival?	1
	4. What is the season now (spring, summer, fall, or winter)?	1
	5. What is the name of this county or district?	1
	6. Please name as many kinds of food as possible in 1 min (1 point for each food and 7 points for those who name 7 or more foods).	7
Registration	7–9. Please repeat these three objects: table, apple, clothes.	3
Attention and calculation	10–14. I will ask you to spend $3 from $20, then you must spend $3 from the number you arrived at and continue to spend $3 until you are asked to stop.	5
	15. I want you to draw the figure on B Card.	1
Recall	16–18. Repeat the three objects learned a little while ago (table, apple, clothes).	3
Language	19–20. Naming pen and watch.	2
	21. Repeat the following sentence: what you plant, what you will get.	1
	22–24. I will give you a piece of paper. You must take the paper using your right hand, fold it in the middle using both hands, and place the paper on the floor.	3

The MMSE is a frequently used 24-item assessment of cognitive functioning. Referring to Zhang (2006), the results of item-6 were evaluated by scoring point for each food and 7 points for those who name 7 or more foods. For the other 23 questions, participants who got the correct answer received a score of 1 (otherwise 0). The total score for the MMSE ranges from 0 to 30, with higher scores indicating better cognitive ability. In this study, cognitive impairment was defined as an MMSE score ≤24, whereas scores >24 indicate no cognitive impairment (Ganguli et al., 2004). Cronbach’s coefficient alpha of scales testing orientation, registration, attention or calculation, recall, and language were 0.756, 0.769, 0.732, 0.754, and 0.729, respectively. The total Cronbach’s coefficient alpha was 0.789, indicating good internal consistency of the items in the scale.

### Sleep measures

To measure sleep duration, we used the following question: “How many hours of actual sleep did you get at night (average hours for one night)?” To further examine any association between sleep duration ranges and cognitive function, participants were divided into short (<6 h), moderate (6–8 h; reference group), and long (>8 h) groups (Li et al., 2022; Ding et al., 2020). According to the results of restricted cubic spline (RCS), we recoded sleep duration as a dichotomous variable (≤7h vs. > 7 h) to better understand the relationship between change in sleep duration and cognitive change.

### Covariates

Referring to Zhang et al. (2021) and Lin et al. (2022), the covariates included sociodemographic variables and risk factors for cognitive function. The sociodemographic variables are age (years), sex (male or female), body mass index (BMI), marital status (married, separated/divorced, and single), household income (RMB), residential area (rural or urban), living arrangement (live with families or live alone), smoking status (non-smoker or smoker), drinker status (non-drinker or drinker), and ADL disability (yes or no). A respondent was defined as ADL disabled if any difficulty in one or more of the above six activities was reported. For each of the following items, score “1” if the respondent had no difficulty in finishing it, or “0” if the respondent had difficulty completing the following tasks: (1) bathing; (2) dressing; (3) toileting; (4) indoor transferring; (5) continence; (6) eating.

Referring to Iizuka et al. (2019) and Han et al. (2022), the risk factors for cognitive function are sleep quality (poor, fair, and good), regular physical activity (yes or no), social activities (never, irregular, and regular), and cultural activities (never, irregular, and regular).

### Statistical analysis

We described the characteristics of participants based on two groups according to the respondent’s MMSE score (cognitively impaired group, cognitively normal group). Basic characteristics are shown as the frequencies and percentages (*N*, %) for categorical variables and mean values for continuous variables. The differences between the two groups were investigated through the Chi-squared test for categorical variables and the *t*-test for continuous variables. The descriptive statistical analysis of all variables is shown in Table 2.

**TABLE 2:** Baseline characteristics of study participants in the CLHLS 2018.

Variables	Total	Cognitive impairment	Normal cognition	*P*-value*^a^*
		
	(*n* = 2,195)	(*n* = 544)	(*n* = 1,651)	
**Sleep duration (hours/per day)**				
Short	417 (19.0%)	119 (21.9%)	298 (18.1%)	0.05
Moderate	1,266 (57.7%)	247 (45.4%)	1,019 (61.7%)	0.001
Long	512 (23.3%)	178 (32.7%)	334 (20.2%)	0.001
Age, years	83.89 ± 9.63	89.15 ± 8.27	82.16 ± 8.03	0.001
≤ 80	848 (38.6%)	87 (16.0%)	761 (46.1%)	0.05
> 80	1,347 (61.4%)	457 (84.0%)	890 (53.9%)	0.001
Sex (males)	1,108 (50.5%)	177 (32.5%)	931 (56.4%)	0.001
BMI (kg/m^2^)	23.18 ± 12.50	22.22 ± 8.38	23.49 ± 13.58	0.001
Marital status (married)	1,212 (55.2%)	392 (72.1%)	820 (49.7%)	0.001
Household income (RMB)	9.83 ± 1.79	9.74 ± 1.95	9.86 ± 1.74	0.001
Residential area (urban)	449 (20.5%)	84 (15.4%)	365 (22.1%)	0.001
Living arrangement (live alone)	414 (18.9%)	101 (18.6%)	313 (19.0%)	0.83
**Sleep quality**				
Poor	317 (14.4%)	92 (16.9%)	225 (13.6%)	0.05
Fair	739 (33.7%)	199 (36.6%)	540 (32.7%)	0.09
Good	1139 (51.9%)	253 (46.5%)	886 (53.7%)	0.001
Smoking status	393 (17.9%)	83 (15.3%)	310 (18.8%)	0.06
Drinker status	378 (17.2%)	60 (11.0%)	318 (19.3%)	0.001
ADL disability	312 (14.2%)	184 (33.8%)	128 (7.8%)	0.001
Regular physical Activity	709 (32.3%)	123 (22.6%)	586 (35.5%)	0.001
**Social activity**				
Never	736 (33.5%)	268 (49.3%)	468 (28.3%)	0.001
Irregular	731 (33.3%)	158 (29.0%)	573 (34.7%)	0.05
Regular	728 (33.2%)	118 (21.7%)	610 (36.9%)	0.001
**Cultural activity**				
Never	1781 (81.1%)	521 (95.8%)	1260 (76.3%)	0.001
Irregular	204 (9.3%)	14 (2.6%)	190 (11.5%)	0.001
Regular	210 (9.6%)	9 (1.6%)	201 (12.2%)	0.001

*^a^T*-test for continuous variables and a chi-square test for categorical variables.

We adopted the logistic model to explore the association between sleep duration and the risk of cognitive impairment. Odds ratios (ORs) and 95% confidence intervals (95% CIs) were calculated to estimate the effects of sleep duration on the risk of cognitive impairment. In addition, further analyses were performed to examine the association between the changes in sleep parameters and cognitive changes. **First,** we examined whether the combination at two-time points affected the risk of cognitive impairment. **Second,** we performed a multinomial logistic regression analysis to estimate the association between sleep duration and cognitive changes over 4 years. Specifically, for the MCI in CLHLS (2014), we classified the outcome at follow-up (CLHLS, 2018) into 3 categories: (1) remained as MCI (18 ≤ MMSE score ≤ 24), (2) reverted to normal cognition (MMSE >24), and (3) progressed to severe cognitive impairment (MMSE <18) (Wang et al., 2010). **Third,** we investigated the association between the change in sleep parameters (7 h as cutoff points) and cognitive changes over 4 years. Statistical analysis was conducted using the R software version 4.1.0.

## Results

### Characteristics of samples

Table 2 represented the comparison between cognitively impaired (MMSE score ≤24) and cognitively normal (MMSE scores >24) by covariates. Among the 2,195 participants, 417 participants (19.0%) slept for less than 6 h, 1,266 participants (57.7%) slept for 6–8 h, and 512 participants (23.3%) slept for 8 h or more. The majority (75.2%) had an MMSE score between 25 and 30 (normal cognition), with the remainder (24.8%) having an MMSE score ≤24(cognitive impairment).

Of all the respondents, there were 1,087 (49.5%) females and 1,108 (50.5%) males. The majority of the participants lived in rural areas (79.5%) and lived with their household members to share in their later years (81.1%). More than half of the elderly had good self-reported sleep quality (51.9%). Few older people reported being current smokers or drinkers, accounting for 17.9 and 17.2%, respectively. Most Chinese seniors didn’t participate in physical activity (67.7 %) or cultural activities (81.1%).

In total, 61.7% of participants with moderate sleep have normal cognition, while only 45.4% of participants with moderate sleep experienced cognitive impairment; this difference is significant (*P* < 0.001). Respondents who suffered from cognitive impairment were more likely to be female, older, married, living in rural areas, living with family, smoker or drinker, have ADL difficulties, and never participate in leisure activities (physical activity, social activity, and cultural activity).

### Associations between sleep duration and cognitive impairment

Results from the unadjusted and covariate-adjusted multilevel models are presented in Table 3. Model 1 was a crude model. Model 2 was adjusted for sociodemographic variables. Model 3 was further simultaneously adjusted by sleep quality, regular physical activity, social activities, and cultural activities. Model 4 was the same as model 3 plus the interaction term between sleep duration and sleep quality.

**TABLE 3:** Impact of sleep duration on the risk of cognitive impairment at follow-up (CLHLS, 2018)^ a^ (*N* = 2,195).

Variables	Model 1	Model 2	Model 3	Model 4
	
	OR (95% CI)	OR (95% CI)	OR (95% CI)	OR (95% CI)
**Sleep duration**				
Short (< 6 h)	1.64 (1.27–2.12)***	1.54 (1.16–2.04)**	1.39 (1.01–1.92)*	0.67 (0.24–1.61)
Moderate (6–8 h)	Ref	Ref	Ref	Ref
Long (>8 h)	2.19 (1.74–2.76)***	1.86 (1.44–2.41)***	1.97 (1.50–2.58)***	1.75 (1.26–2.43)***
**Duration × Quality**				
Short*Fair				2.91 (1.08–8.63)*
Short*Bad				1.70 (0.57–5.49)
Long* Fair				1.38 (0.74–2.56)
Long* Bad				1.35 (0.42–4.43)
Age (>80)		3.12 (2.39–4.12)***	2.87 (2.18–3.81)***	2.89 (2.20–3.84)***
Sex (males)		0.44 (0.34–0.56)***	0.51 (0.40–0.67)***	0.52 (0.40–0.67)***
BMI (kg/m^2^)		0.99 (0.97–1.00)	0.99 (0.97–1.00)	0.99 (0.97–1.00)
Marital status (married)		1.39 (1.06–1.81)*	1.37 (1.05–1.80)*	1.36 (1.04–1.78)*
Household income (RMB)		0.96 (0.90–1.02)	0.97 (0.91–1.03)	0.97 (0.91–1.03)
Residential area (urban)		0.49 (0.36–0.66)***	0.62 (0.44–0.86)**	0.61 (0.44–0.85)**
Living arrangement (live alone)		0.80 (0.59–1.07)	0.84 (0.62–1.14)	0.85 (0.62–1.14)
Smoking status		1.43 (1.03–1.97)*	1.46 (1.04–2.03)*	1.47 (1.05–2.05)*
Drinker status		0.81 (0.57–1.04)	0.84 (0.58–1.18)	0.83 (0.58–1.18)
ADL disability		4.74 (3.59–6.03)***	3.92 (2.92–5.29)***	4.01 (2.98–5.41)***
**Sleep quality**				
Poor			1.18 (0.83–1.68)	0.85 (0.23–3.20)
Fair			Ref	Ref
Good			0.86 (0.66–1.22)	0.26 (0.07–0.80)*
Regular physical activity			0.91 (0.70–1.19)	0.92 (0.70–1.20)
**Social activity**				
Never			Ref	Ref
Irregular			0.87 (0.66–1.14)	0.87 (0.66–1.14)
Regular			0.56 (0.42–0.75)***	0.56 (0.42–0.75)***
**Cultural activity**				
Never			Ref	Ref
Irregular			0.31 (0.16–0.54)***	0.31 (0.16–0.54)***
Regular			0.21 (0.09–0.41)***	0.21 (0.09–0.41)***

*^a^*Multiple logistic regression.

*p < 0.05, **p < 0.01, ***p < 0.001.

In Model 3, short or long sleep duration was significantly associated with a higher risk of cognitive impairment, with moderate sleep durations as the reference (column 4 of Table 3). In Model 4, short sleep durations with fair sleep quality had a higher risk of cognitive impairment (OR = 2.91, 95% CI: 1.08–8.63). The subgroup analyses show that the elderly who are older, female, married, smoking, live in the rural area, have ADL difficulties, and never participate in social activities or cultural activities predict an increased risk of cognitive decline than their counterparts (column 4 of Table 3).

**FIGURE 2:**
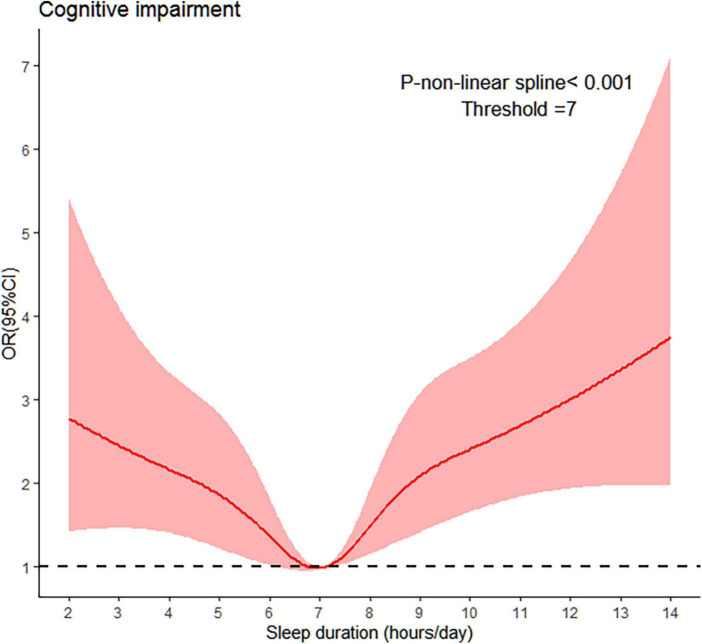
Associations between sleep duration and cognitive impairment using a RCS regression. Using logistics regression to get the odds ratio after adjusting for age, sex, body mass index (BMI), marital status, household income (RMB), residential area, living arrangement, smoking status, drinker status, ADL disability, sleep quality, regular physical activity, social activities, and cultural activities.

We further evaluated the relationship between sleep duration and cognitive impairment by RCS. The results showed a U-shaped relationship between sleep duration and risk of cognitive impairment (Figure 2).

Table 4 presents the adjusted associations between sleep duration and cognitive impairment of persons with normal cognition at baseline. Those who slept > 7 h at follow-up (OR, 1.28; 95% CI, 1.00–1.71) were at an increased risk of cognitive impairment, whereas baseline sleep duration had shown insignificant effects. The individual’s sleep duration > 7 h at both baseline and follow-up period (OR, 1.50; 95% CI, 1.00–2.31) had an approximately 1.5 times higher risk of cognitive decline.

**TABLE 4:** Associations between changes in sleep duration across 2014 and 2018 and cognitive impairment*^a^*.

Normal cognition at baseline (*n* = 1,851)	Cognitive impairment	
	No. Events/Total	OR (95% CI)
**Sleep duration at baseline, h**		
≤7	175/953	Ref
> 7	170/898	1.16 (0.89–1.52)
**Sleep duration at follow-up, h**		
≤7	180/1045	Ref
> 7	165/806	1.28 (1.00–1.71)*
**Changes in sleep duration over 4 years, h**		
≤7 at both baseline and follow-up	107/610	Ref
Changed from ≤7 to > 7	68/343	1.09 (0.72–1.64)
Changed from > 7 to ≤7	73/435	0.66 (0.43–1.01)
>7 at both baseline and follow-up	97/463	1.50 (1.00–2.31)*

*^a^*Multiple logistic regression analysis adjusted for age, sex, body mass index (BMI), marital status, household income (RMB), residential area, living arrangement, smoking status, drinker status, ADL disability, sleep quality, regular physical activity, social activities, and cultural activities.

*p < 0.05.

### Associations between changes in sleep duration and cognitive changes over 4 years

Among the 259 participants with MCI (18 ≤ MMSE score ≤ 24) at baseline, 126 individuals transitioned to normal cognition and 52 into severe cognitive impairment. As shown in Table 5, those sleeping > 7 h at follow-up (OR, 0.46; 95% CI, 0.22–0.96) had about a 54% lower chance of reverting to normal cognition. In addition, those who sleeping > 7 h at baseline and 4-year follow-up assessments (OR, 0.25; 95% CI, 0.09–0.66) had about a 75% lower chance of reverting to normal cognition, respectively (Table 5). However, sleep duration at baseline had no association with cognitive change during 4-year period.

**TABLE 5:** Impact of change of sleep parameters on the cognitive changes over 4 years*^a^*.

MCI at baseline (*n* = 259)	Reverted to normal cognition		Progressed to severe cognitive impairment	
	No. Events/Total	OR (95% CI)	No. Events/Cognitive Total	OR (95% CI)
**Sleep duration at baseline, h**				
≤7	72/133	Ref	24/133	Ref
> 7	54/126	0.54 (0.28–1.03)	28/126	0.98 (0.45–2.15)
**Sleep duration at follow-up, h**				
≤7	79/140	Ref	21/140	Ref
> 7	47/119	0.46 (0.22–0.96)*	31/119	1.26 (0.51–3.07)
**Changes in sleep duration over 4 years, h**				
≤7 at both baseline and follow-up	46/80	Ref	13/80	Ref
Changed from ≤7 to >7	26/53	0.54 (0.20–1.50)	11/53	0.85 (0.24–3.03)
Changed from >7 to ≤7	33/60	0.62 (0.25–1.46)	8/60	0.65 (0.20–2.11)
>7 at both baseline and follow-up	21/66	0.25 (0.09–0.66)**	20/66	1.12 (0.36–3.47)

*^a^*Multinomial logistic regression analysis adjusted for age, sex, body mass index (BMI), marital status, household income (RMB), residential area, living arrangement, smoking status, drinker status, ADL disability, sleep quality, regular physical activity, social activities, and cultural activities.

*p < 0.05, **p < 0.01.

### Sensitivity analyses

Referring to Xiong et al. (2022), this study uses a machine learning method, the Random Forest (RF) algorithm, to interpolate all the missing values of covariates. As shown in Table 6, the estimation results of sleep duration on cognitive impairment are similar to the results of Table 3, indicating that this study has good robustness.

**TABLE 6:** Sensitivity analyses including the interpolated missing values of covariates^a^ (*N* = 2,635).

Variables	Model 1	Model 2	Model 3	Model 4
	
	OR (95% CI)	OR (95% CI)	OR (95% CI)	OR (95% CI)
**Sleep duration**				
Short (< 6 h)	1.71 (1.35–2.15)***	1.58 (1.22–2.04)***	1.37 (1.02–1.83)*	0.53 (0.21–1.19)
Moderate (6–8 h)	Ref	Ref	Ref	Ref
Long (> 8 h)	2.21 (1.35–2.15)***	1.80 (1.43–2.27)***	1.94 (1.52–2.48)***	1.71 (1.28–2.30)***
**Duration × Quality**				
Short*Fair				3.69 (1.49–10.02)**
Short*Bad				2.21 (0.81–6.51)
Long* Fair				1.40 (0.79–2.45)
Long* Bad				1.16 (0.40–3.37)

^a^Models were adjusted as we did in Table 3.

*p < 0.05, **p < 0.01, ***p < 0.001.

## Discussion

In this study, we explored the influence of sleep duration on cognitive impairment, as well as the longitudinal association between the changes in sleep duration and cognitive change with a 4 years follow-up from 2014 to 2018. Older adults with cognitive impairment and normal cognition were 24.8 and 75.2%, respectively. Compared with moderate sleep duration between 6 and 8 h, short (<6 h) and long (>8 h) sleep durations had a stronger association with cognitive impairment. Similar associations were also found in other studies (Ma et al., 2020; Fu et al., 2021). Several possible explanations for their relationship can be suggested. First, short durations of sleep would lead to elevated stress hormones (Reynolds et al., 2010), which had been linked with cognitive decline. Second, longer sleep durations could contribute to elevated levels of inflammatory markers (Irwin et al., 2016) and psychiatric disorders (i.e., either anxiety or depression) (Zhai et al., 2015; Dong et al., 2022), which in turn can lead to cognitive decline. Also, short sleep durations with fair sleep quality were detrimental to cognitive performance. Short sleep duration will lead to circadian dysfunction, and circadian dysfunction was associated with cognitive decline (Xiong et al., 2021a).

In our study, participants living in rural areas have an increased risk of cognitive decline than participants living in urban areas. The result was in line with some previous existing literature (Jia et al., 2014; Xiong et al., 2021b). This may be because, under the urban-rural dual structure, rural areas have relatively few entertainment products and local services compared with urban areas. Participation in social activities and cultural activities might significantly contribute to prevention of cognitive decline and dementia, this was consistent with the study of Fancourt et al. (2018). Probably because social activities could not only reduce perceived isolation but minimize the negative impacts of sedentary behaviors by encouraging older adults to leave their homes. Cultural participation (i.e., reading activities) activated several cognitive processes, such as working memory capacity (Baddeley, 2003), executive functioning (Swanson and O’Connor, 2009), and the ability of decoding (Perfetti, 1985).

Few previous studies have focused on the relationship between the changes in sleep duration and cognitive changes. In this longitudinal study, we explored the association between changes in sleep duration and cognitive change. Our findings unveiled that sleeping > 7 h at follow-up period was associated with cognitive impairment in both those with normal cognition and MCI at baseline. However, Westwood et al. (2017) demonstrated that prolonged sleep duration was only associated with the risk of cognitive decline in persons with MCI, but not with normal cognition. Amyloid β, a key mechanism in the development of Alzheimer’s disease pathology, begins to accumulate before an individual is diagnosed with MCI (Holth et al., 2017). Those with MCI would have some psychiatric disorders such as anxiety and depression (Gabryelewicz et al., 2004), which are closely related to sleep disturbances. This might explain our findings.

In addition, sleeping > 7 h at both baseline and 4-year follow-up assessments was associated with cognitive impairment in both those with normal cognition and MCI at baseline. Several previous studies supported our findings. For example, Suh et al. (2018) conducted a cross-sectional study and found that long sleep duration at both the baseline and 4-year follow-up assessments had a high risk of cognitive decline among the Korean elderly, while no significant association was detected between an increase sleep duration from short to long and cognitive change.

However, our study also had certain limitations that should be mentioned. Firstly, the measures of sleep duration were calculated based mainly on self-reported and single-item, which could be biased. Nevertheless, plenty of large cohorts confirmed the identified relationship between self-reported sleep duration and objectively ascertained health outcomes, making our research more reliable and convincing (Jike et al., 2018; Kwok et al., 2018). Secondly, of all the respondents, 1,525 were lost to follow up and 2,226 died in CLHLS (2018), which accounted for 21.2 and 31.0% of the total included participants, respectively. This might affect our result. Thirdly, the CLHLS did not collect the sleep-related parameters that might impact cognitive performance, such as sleep efficiency, midsleep time, sleep latency, and sleep efficiency. Therefore, further research is necessary to confirm the relationship between sleep-related parameters and cognitive function to find more effective ways to prevent sleep problems and cognitive impairment.

## Conclusion

The identified relationship in this study would provide further evidence to determine the optimal sleep duration in the elderly. For those with long sleep durations, policies and interventions that target cognitive impairment should emphasize the importance of normal sleep durations and avoid excessively long sleep durations. This would be important to offset the increasing burdens to individuals and society arising from late-life dementia and cognitive impairment in our rapidly aging populations.

## Data availability statement

Publicly available datasets were analyzed in this study. This data can be found here: https://opendata.pku.edu.cn/.

## Author contributions

W-CC conceptualized the manuscript and designed the methodology and writing—original draft preparation. W-CC and X-YW contributed to the data analysis and writing—review and editing. Both authors have read and agreed to the published version of the manuscript.
